# Long‐term SARS‐CoV‐2‐specific and cross‐reactive cellular immune responses correlate with humoral responses, disease severity, and symptomatology

**DOI:** 10.1002/iid3.595

**Published:** 2022-03-14

**Authors:** Ida Laurén, Sebastian Havervall, Henry Ng, Martin Lord, Aleksandra Pettke, Nina Greilert‐Norin, Lena Gabrielsson, Aikaterini Chourlia, Catarina Amoêdo‐Leite, Vijay S. Josyula, Mohamed Eltahir, Iliana Kerzeli, August J. Falk, Jonathan Hober, Wanda Christ, Anna Wiberg, My Hedhammar, Hanna Tegel, Joachim Burman, Feifei Xu, Elisa Pin, Anna Månberg, Jonas Klingström, Gustaf Christoffersson, Sophia Hober, Peter Nilsson, Mia Philipson, Pierre Dönnes, Robin Lindsay, Charlotte Thålin, Sara Mangsbo

**Affiliations:** ^1^ Department of Pharmacy, Science for Life Laboratory Uppsala University Uppsala Sweden; ^2^ Department of Clinical Sciences Karolinska Institute, Danderyd Hospital Stockholm Sweden; ^3^ Department of Medical Cell Biology, Science for Life Laboratory Uppsala University Uppsala Sweden; ^4^ Department of Oncology‐Pathology Karolinska Institute Stockholm Sweden; ^5^ Department of Immunology, Genetics, and Pathology Uppsala University Uppsala Sweden; ^6^ Division of Affinity Proteomics, Department of Protein Science KTH Royal Institute of Technology, Science for Life Laboratory Stockholm Sweden; ^7^ Department of Medicine Huddinge Karolinska Institute, Centre for Infectious Medicine Stockholm Sweden; ^8^ Division of Protein Technology, Department of Protein Science KTH Royal Institute of Technology Stockholm Sweden; ^9^ Department of Neuroscience Uppsala University Uppsala Sweden; ^10^ SciCross AB Skövde Sweden

**Keywords:** B‐cell, IFNγ, IL‐2, SARS‐Cov‐2, T cell

## Abstract

**Background:**

Cellular immune memory responses post coronavirus disease 2019 (COVID‐19) have been difficult to assess due to the risks of contaminating the immune response readout with memory responses stemming from previous exposure to endemic coronaviruses. The work herein presents a large‐scale long‐term follow‐up study investigating the correlation between symptomology and cellular immune responses four to five months post seroconversion based on a unique severe acute respiratory syndrome coronavirus 2 (SARS‐CoV‐2)‐specific peptide pool that contains no overlapping peptides with endemic human coronaviruses.

**Methods:**

Peptide stimulated memory T cell responses were assessed with dual interferon‐gamma (IFNγ) and interleukin (IL)‐2 Fluorospot. Serological analyses were performed using a multiplex antigen bead array.

**Results:**

Our work demonstrates that long‐term SARS‐CoV‐2‐specific memory T cell responses feature dual IFNγ and IL‐2 responses, whereas cross‐reactive memory T cell responses primarily generate IFNγ in response to SARS‐CoV‐2 peptide stimulation. T cell responses correlated to long‐term humoral immune responses. Disease severity as well as specific COVID‐19 symptoms correlated with the magnitude of the SARS‐CoV‐2‐specific memory T cell response four to five months post seroconversion.

**Conclusion:**

Using a large cohort and a SARS‐CoV‐2‐specific peptide pool we were able to substantiate that initial disease severity and symptoms correlate with the magnitude of the SARS‐CoV‐2‐specific memory T cell responses.

## Graphical abstract

A strong correlation between interferon‐gamma (IFNγ) and interleukin‐2) (IL‐2) producing SARS‐CoV‐2‐specific memory T cell responses four months post mild to severe COVID‐19. Less cross‐reactive responses with a novel designed peptide pool (4%). Cross‐reactive T cell secretion in repeatedly seronegative individuals was IFNγ‐dominant. Four specific COVID symptoms correlated with the magnitude of the cellular response. 

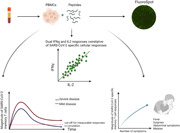

## 1 INTRODUCTION

Understanding long‐term immune responses after severe acute respiratory syndrome coronavirus 2 (SARS‐CoV‐2) infection is key to reduce the widespread global effects on both health and society as a whole, and can also guide vaccination strategies. The vast majority of SARS‐CoV‐2‐infected individuals seroconvert,^1^, ^2^ and there is strong evidence for long‐lasting circulating neutralizing antibodies after both severe and mild coronavirus disease 2019 (COVID‐19).^3^, ^4^, ^5^ In addition, SARS‐CoV‐2 has been shown to induce effector T cell responses,^6^ reduce circulating numbers of CD4^+^ and CD8^+^ T cells^6^, ^7^, ^8^ and cause immune misfiring during acute infection.^9^ However, while most recovered individuals present a measurable and lasting immunity, there are also case reports of reinfected individuals.^10^, ^11^, ^12^

**Figure 1:**
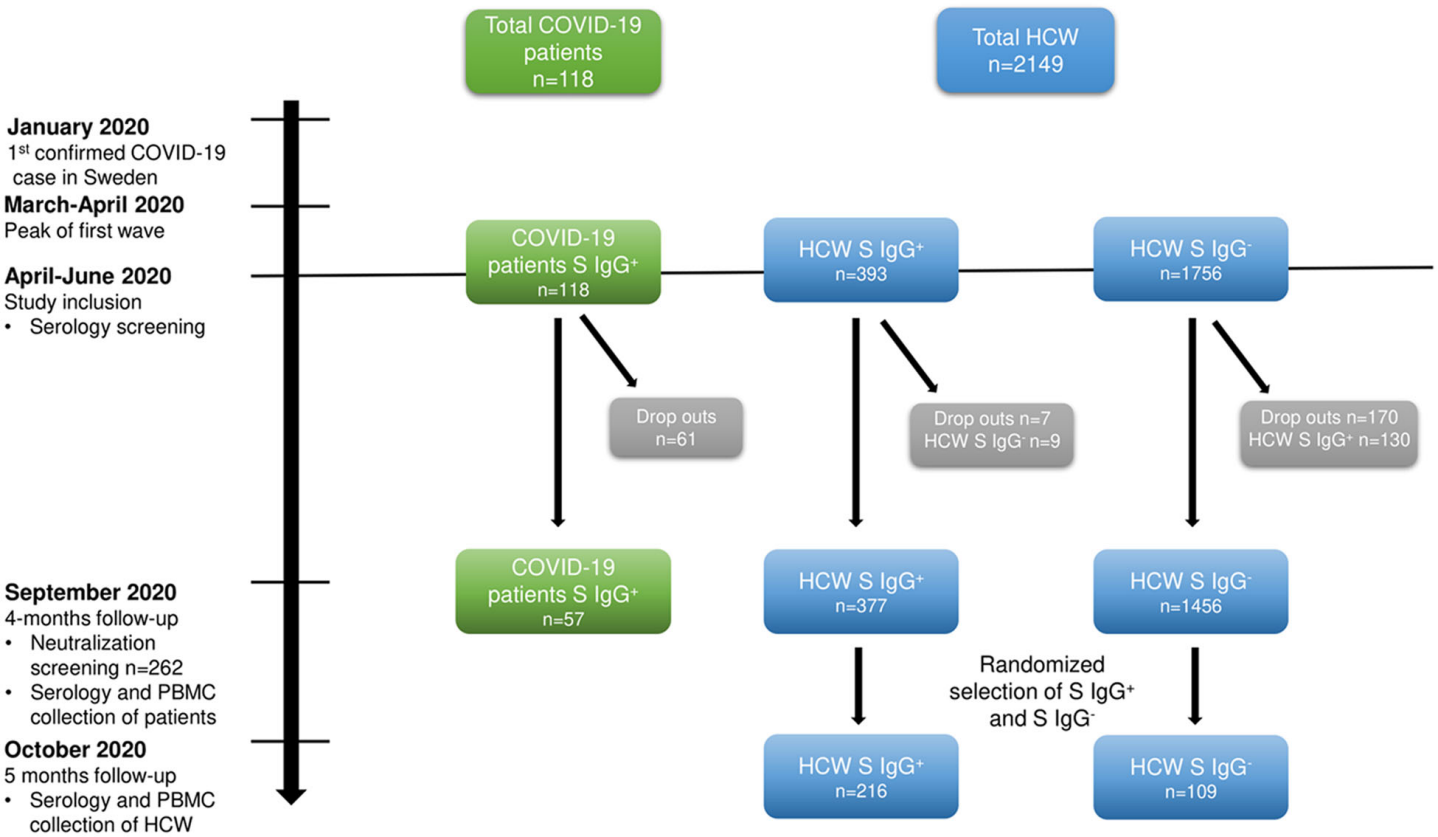
Study timeline and cohort selection. Timeline of study sample collection and testing. COVID‐19 patients (*n* = 118) and HCW (*n* = 2149) were tested for serological status (anti‐Spike IgG) in April‐May 2020 following the first wave of SARS‐CoV‐2 infections in Sweden. At the 4 months follow‐up in September 2020, blood samples were collected for serology in the whole cohort, for neutralizing antibodies in a subgroup (57 patients and 205 HCW) and PBMC were collected from the convalescent patients only. PBMC was collected from a subgroup HCW in October 2020. HCW who had developed SARS‐CoV‐2‐specific antibodies between the time points were excluded from this subgroup and the remaining HCW (IgG + *n* = 377, IgG−*n* = 1456) were randomly selected for PBMC sampling (IgG + *n* = 216, IgG−*n* = 109) at the 5 months follow‐up. COVID‐19, coronavirus disease 2019; HCW, healthcare worker; PBMC, peripheral blood mononuclear cells; SARS‐CoV‐2, severe acute respiratory syndrome coronavirus 2

In veterinary medicine, it has been reported that coronaviruses show poor cross‐reactive protective immunity between serotypes^13^, ^14^, ^15^ and that some coronaviruses can cause persistent infections.^16^, ^17^ During the current pandemic, multiple publications have shown that pre‐existing memory T cell responses to SARS‐CoV‐2 peptides are present, possibly from previous exposure to endemic human coronaviruses (HCoV)−229E, ‐NL63, ‐OC43, and ‐HKU1 are measurable in unexposed individuals.^18^, ^19^, ^20^, ^21^ However, the clinical significance of a pre‐existing and cross‐reactive T cell memory response is under debate and poses a challenge in investigations of SARS‐CoV‐2‐specific memory T cells. We herein explored the relationship between humoral responses, SARS‐CoV‐2 exposure, symptomatology, and cellular immune memory responses in an ongoing longitudinal cohort study.^22^, ^23^, ^24^ In this study, blood samples were obtained from 216 healthcare workers (HCW) five months post‐SARS‐CoV‐2 spike (S) IgG seroconversion and 115 HCW who had been SARS‐CoV‐2 S IgG seronegative at repeated occasions during the study period (Figure 1). Blood samples were also obtained from 57 COVID‐19 patients 4 months post severe disease and SARS‐CoV‐2 S IgG seroconversion. HCW were stratified according to anti‐S IgG seroconversion, self‐reported symptoms during the acute infection, and SARS‐CoV‐2 neutralizing antibodies. To enable the investigation of SARS‐CoV‐2‐specific memory T cell responses, we designed a peptide pool comprising only confirmed SARS‐CoV‐2‐specific peptides. T cells were stimulated with the designed peptide pool and a commercial SARS‐CoV‐2 peptide pool that includes an identified immunodominant epitope overlapping with endemic HCoVs. Overall, our data demonstrate that long‐term SARS‐CoV‐2‐specific memory T cell responses are long‐lasting, associated with an effective and durable antibody response, and correlate with disease severity.

**Table 1:** Demographics, symptomatology, and serology status

Healthcare workers	All	Seropositive	Seronegative
*n* (%)	325 (100)	216 (65)	109 (35)
Age, median (IQR)	44 (34–53)	46 (36–54)	44 (35–53)
Female, *n* (%)	280 (86)	184 (85)	96 (88)
Male, *n* (%)	45 (14)	32 (15)	13 (12)
Symptoms before inclusion, *n* (%)			
Fever	159 (49)	124 (57)	35 (32)
Headache	182 (56)	133 (62)	49 (45)
Anosmia	142 (44)	117 (54)	25 (23)
Ageusia	134 (41)	109 (50)	25 (23)
Cough	139 (43)	109 (50)	30 (28)
Malaise	172 (53)	133 (62)	39 (36)
Abdominal symptoms	93 (30)	67 (31)	26 (24)
Sore throat	127 (39)	75 (35)	52 (48)
Shortness of breath	61 (19)	47 (22)	14 (13)
Patients (all seropositive)	All		
*n* (%)	57 (100)		
Demographics			
Age, median (IQR)	59 (48–66)		
Female, *n* (%)	17 (30)		
Male, *n* (%)	40 (70)		
Level and duration of hospital stay			
ICU, *n* (%)	5 (9)		
Intermediate ward, *n* (%)	5 (9)		
General ward, *n* (%)	47 (82)		

Abbreviations: ICU, intensive care unit; IQR, interquartile range.

## 2 METHODS

### 2.1 Data availability

The datasets supporting the current study have not been deposited in a public repository because of legal and privacy obligations but are available from the corresponding author on request.

### 2.2 Study population

The COMMUNITY study (COVID‐19 Immunity Study, Dnr 2020‐01653) is a longitudinal study investigating long‐term immunity after COVID‐19. Two thousand one hundred and forty‐nine HCW and 118 hospitalized COVID‐19 patients were included at Danderyd hospital, Stockholm, Sweden in April–June 2020. During this time, there were no known SARS‐CoV‐2 variants circulating in Sweden, and no individuals included in the study had been vaccinated as this study was performed prior vaccine approval in Sweden. Blood samples were collected at study inclusion and prospectively every four months (Figure 1). The study population and hospital setting have been described elsewhere.^22^, ^23^, ^24^, ^44^, ^45^ Briefly, COVID‐19 patients were diagnosed by reverse‐transcriptase polymerase chain reaction (PCR) viral detection of oropharyngeal or nasopharyngeal swabs. Exclusion criteria were age <18 years or non‐consent to participate in the study. Demographic and clinical data on the patient group were obtained from medical journals (Table 1). PCR viral detection was not available for HCW before study inclusion, but the cohort has been profiled for serology against multiple SARS‐CoV‐2 antigens both at inclusion and at regular follow‐ups. All HCW completed a questionnaire at the time of each blood sampling, comprising self‐reported predefined symptoms experienced before blood sampling and compatible with COVID‐19 (fever, headache, anosmia, ageusia, cough, malaise, common cold, abdominal pain, sore throat, shortness of breath, and joint/muscle pain) and occupation. For this sub‐study, a subset of blood samples from 57 COVID‐19 patients was obtained from the four months follow‐up visit (4 months posthospitalization [mean 4.2 months, *SD* 0.52]). Only serum samples were collected from the HCW at the four months follow‐up. New blood samples were therefore collected for this sub‐study in a 5‐month follow‐up (mean 5.3, *SD* 0.27). HCW were stratified according to SARS‐CoV‐2 serology (anti‐S IgG), see Figure 1. The majority of HCW were women (*n* = 285, 86%) and the mean age was 44 (SD 12) years. The majority of anti‐S IgG positive HCW (90%, 194/216) reported mild symptoms before study inclusion, and 9% (20/216) had been asymptomatic. The patient group comprised a majority of men, 70% (40/57), and the mean age was 57 (*SD* 14) years. 18% (10/57) had required intermediate or intensive care, while 82% (47/57) had been admitted to general wards during the acute COVID‐19 infection. The study was approved by the Swedish Ethical Review Authority and informed consent was obtained from all study participants.

### 2.3 Selection of peptides from literature and in silico analysis

A peptide pool was designed using a combination of published data and in silico analysis of SARS‐CoV‐2 B/T cell epitopes enabling the assessment of specific T cell responses. Structural information was utilized to identify exposed structures of the spike RBD (receptor binding domain).^46^ The complete SARS‐CoV‐2 genome was scanned for potential T cell epitopes using the SciCross AB (Skövde) Immunogenicity Platform (SCIP) in silico algorithms. Identified T and B cell epitopes were further collected from publications reporting their immunogenicity.^19^, ^20^, ^26^ In addition, the genomic variation of the SARS‐CoV‐2 virus was considered a long with the wild‐type sequence^47^, ^48^ and both the NP‐P13L (peptide 12) as well as the Spike‐D614G (peptide 6) variants were included in the in‐house designed peptide pool. An additional selection criterion was for peptides that bind a wide range of HLA molecules, covering the majority of the population. The final selection of peptides was based on verified activation of B or T cells from literature, as well as predicted promiscuous T cell epitopes (covering both HLA class I and HLA class II).

The in‐house generated SARS‐CoV‐2‐specific peptide pool (TS16) consists of 16 peptides (Table S1) covering the SARS‐CoV‐2 spike (S), nucleocapsid protein (N), membrane protein (M), and open reading frame (ORF) 3 and 7 with a purity of > 95%, also used in Havervall et al.^23^ and Mangsbo et al.^25^ The commercial peptide pool covering multiple SARS‐CoV‐2 antigens and an immunodominant epitope overlapping with endemic HCoVs (TB47) was the S, N, M, and O defined peptide pool purchased from Mabtech AB (Stockholm, Sweden) and includes 47 peptides with a mean purity of 80% (60%–99%) (Table S2).

### 2.4 Description of in silico prediction of T cell epitopes and population coverage

Peptides were selected for T cell epitopes using in silico prediction. Both CD4^+^ and CD8^+^ epitopes were assessed using HLA class I and HLA class II peptide binding prediction to identify nine amino acid long sequences with HLA binding cores for potential T cell epitopes. HLA alleles were selected to give a broad coverage of the general population (HLA Class I: A*02:01, A*01:01, A*03:01, A*11:01, A*24:02, B*07:02, B*08:01, and B*40:01; HLA Class II: DRB1*01:01, DRB1*03:01, DRB1*04:01, DRB1*07:01, DRB1*08:01, DRB1*11:01, DRB1*13:01, and DRB1*15:01) and predictions were run using SciCross AB (Skövde) in‐house algorithms. For HLA Class I prediction, a support vector machine (SVM)‐based approach similar to the one described by Dönnes et al. was used.^49^, ^50^ HLA class II prediction was based on both position‐specific matrices and SVM models. Models for HLA Class I and Class II prediction were trained on data from both affinity measurements and naturally eluted HLA ligands.

The population coverage of the peptide pool was calculated based on predicted T cell epitope content. An HLA allele is considered to be covered if at least one T cell epitope is predicted among all peptides of the pool. The population coverage was estimated as described by Bui et al.^51^ This gives a coverage of 88% for HLA Class I and 77% for HLA Class II in a worldwide population (Figure S1A,B).^52^ The combined HLA class I and HLA class II coverage reaches 97% of the population (Figure S1C)

### 2.5 Peptide pool design and epitope cross‐reactivity evaluation

Potential cross‐reactivity towards endemic HCoV was assessed using sequence searches. Identical peptides of different lengths (five amino acids and longer) between selected peptides and HCoV were identified. The shortest length of linear B‐cell epitopes is often considered four to five amino acids in literature,^53^ whereas, for T cell epitopes, an HLA‐binding core of eight‐nine amino acids is more relevant.^54^ Furthermore, a search for T cell epitope matches based on TCR‐facing amino acids was also performed, similar to the method described by Moise et al.^55^ The similarity of two nine amino acid long HLA Class II binding peptides is given by positions two, three, five, seven and eight of the sequences. The amino acids of the other position in the peptide are mainly interacting with the HLA molecule itself, not providing any specificity to the HLA‐peptide:TCR interaction. Reference proteomes used in this analysis, referenced by UniProt proteome IDs, were: UP000145724 (NL63), UP000007552 (OC43), UP000122230 (HKU1), and UP000006716 (229E).

### 2.6 PBMC sampling

White blood cell count (WBC) and lymphocyte count of whole blood were analyzed using a hematology analyser XP‐300 (Sysmex). Blood was drawn in lithium‐heparinized tubes and processed within 24 h. The peripheral blood mononuclear cells (PBMC) isolation was performed using a density gradient with SepMate tubes according to manufactures instructions (StemCell). Briefly, the blood was mixed 1:1 with phosphate‐buffered saline (PBS) (Biowest) before being applied to the SepMate tubes containing Ficoll‐Paque premium (Cytiva). The tubes were centrifuged at 1200×*g* for 10 min at room temperature (RT). The top layer was poured off and washed with PBS following centrifugation at 300×*g* for 8 min at RT. An additional wash was performed with PBS following the last centrifugation of 200×*g* for 5 min at RT before frozen down in fetal bovine serum (FBS) (Gibco) with 10% dimethyl sulfoxide (DMSO) (Tocris).

### 2.7 Fluorospot analysis

SARS‐CoV‐2‐specific T cell reactivity was evaluated by IFNγ and IL‐2 fluorospot (Mabtech) with stimulation of TB47 or TS16 pools as described above. A cytomegalovirus (CMV) specific peptide pool with 42 peptides (Mabtech) was used as a reference to evaluate the antiviral response across the cohort. Anti‐CD3 (CD3‐2 Mabtech) was used as a positive control and DMSO as a negative control. The cryopreserved PBMCs were thawed and rested overnight in complete medium containing RMPI GlutaMax (Gibco) medium supplemented with 10% FBS (Gibco) and 100 Units of Penicillin‐Streptomycin (Gibco). Pre‐coated fluorospot plates with IFNγ (1‐D1K, Mabtech) and IL‐2 (MT2A91/2C95, Mabtech) were washed three times with PBS and blocked with complete medium overnight at 4°C. The cells were harvested and plated in duplicates with 2.5 × 10^5^ cells/well for peptide stimulation, 1 × 10^5^ cells/well for CMV control, and 0.50 × 10^5^ cells/well for anti‐CD3 stimulation. SARS‐CoV‐2‐specific peptide pools or CMV‐specific peptides were added at a concentration of 2 µg/ml for each individual peptide. The cells were stimulated for 24 h at 37°C with 5% CO_2_. The plates were washed five times with PBS before incubated with diluted 1:200 anti‐IFNγ (7‐B6‐1‐BAM) and 1:500 anti‐IL‐2 (MT8G10‐biotinylated) antibodies for 2 h at RT, followed by 1 h incubation with secondary fluorophore‐conjugated antibodies, anti‐BAM 490 (1:200), and streptavidin‐550 (1:200). Lastly, the fluorophore enhancer was added for 10 min. Between each step, the plates were washed five times with PBS, except after the addition of the fluorophore enhancer. The plates were read using a Mabtech IRIS and spots were analyzed using Mabtech Apex software 1.1. SFU/million cells are reported as the peptide stimulated value minus the background unstimulated control. To group positive and negative responders, the threshold was set for a binary T cell response criterion based on a two‐fold or more increase in the spot‐forming units (SFU) above its own negative control value. Individuals with a negative control SFU value below 10 were only scored positive if they had a peptide‐induced memory T cell response that was > the negative control +10. HCW or patients with a negative control sample displaying an SFU higher than the mean for the entire study + 4 SD were excluded.

### 2.8 Serology analyses

Serological analysis was performed using a multiplex antigen bead array in a high throughput 384‐plates format as previously described.^22^, ^56^ IgG reactivity was measured towards spike trimers comprising the perfusion‐stabilized S‐glycoprotein ectodomain (in‐house produced, expressed in HEK, and purified using a C‐terminal Strep II tag) and the C‐terminal domain of the N‐protein (in‐house produced, expressed in Escherichia coli and purified using a C‐terminal His‐tag). The two viral proteins were linked to the surface of color‐coded magnetic microbeads (Luminex Corp) to generate the bead array, and the specific IgG reactivity was detected by means of a phycoerythrine‐conjugates goat anti‐human IgG (H10104, Invitrogen) and measured as mean fluorescent intensity (MFI) in a FlexMap3D system (Luminex Corp). The antigen‐specific threshold for seropositivity was defined as the mean MFI plus 6 SD of 12 negative controls included for each assay run.

### 2.9 Virus neutralization assay

Micro‐neutralization assay was performed on blood samples collected at the four months follow‐up as previously described.^57^ Briefly, serum was heat inactivated and 10‐fold diluted in duplicate. Each dilution was mixed with tissue culture of SARS‐CoV‐2 and incubated. The cells were inspected for signs of cytopathogenic effect (CPE) by optical microscopy after four days. If <50% of the cell layer showed signs of CPE, the well was scored as neutralizing.

### 2.10 Statistical calculations

Statistical analyses were performed in Prism 9 (GraphPad). Data set normality was determined using the Anderson–Darling test, D'Agostino and Pearson test, and Shapiro–Wilk test. Statistical comparisons were performed using Mann–Whitney or Kruskal–Wallis with Dunn's correction for multiple comparisons. The correlation statistical analysis was determined by the two‐tailed Spearman coefficient.

To determine the correlation between symptoms and memory T cell responses, Fisher's exact test was used to calculate the odds ratio (OR) with a 95% confidence interval (CI).

## 3 RESULTS

### 3.1 Alternative peptide pool stimulation reveals a linear relationship between IFNγ and IL‐2 responses in SARS‐CoV‐2‐specific memory T cells, and an IFNγ biased response in cross‐reactive memory T cells

**Figure 2:**
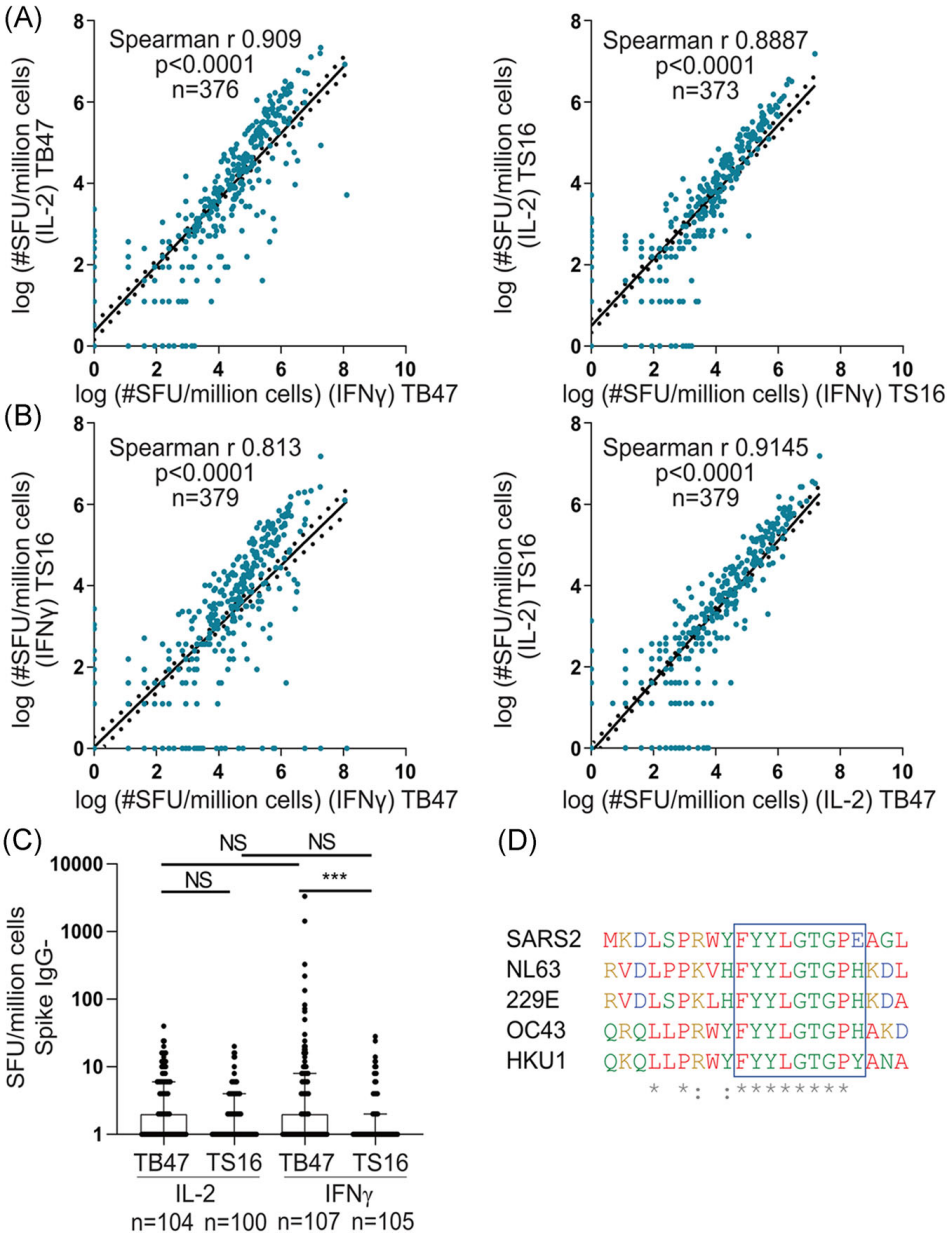
Activation of cross‐reactive T cells selectively triggers IFNγ production. (A) HCW and patient PBMCs stimulated with TB47 (left) or TS16 (right). IFNγ and IL‐2 Spot forming units (SFU) per million cells measured by Fluorospot. Linear regression with 95% confidence intervals displayed. (B) HCW and patients PBMCs stimulated with TB47 or TS16. IFNγ (left) and IL‐2 (right) SFU/million cells measured by Fluorospot. regression with 95% confidence intervals displayed. (C) IL‐2 and IFNγ SFU/million cells from HCW seronegative for Spike IgG at all time‐points. (D) An alignment of a conserved peptide sequence of endemic human coronaviruses with SARS‐CoV‐2. The boxed region highlights a nine amino acid (AA) sequence that represents the TCR exposed residues. “*” indicates conserved residues with exact overlap to the SARS‐CoV‐2 sequence, and “:” indicates conservation of the amino acid groups. Red = small and hydrophobic AA, Blue = Acidic AA, Yellow = Basic‐H AA, Green = Hydroxyl + sulfhydryl + amine + G AA. (A and B) see graphs for n, statistics and the correlations were determined by Spearman r with a 95% confidence interval. Values were transformed with log(x + 1). (C) Median + /IQr. See graphs for n, Kruskal–Wallis test and Dunn's test for multiple comparisons. NS = non significant ***p < .001. HCW, healthcare worker; IFN‐γ, interferon gamma; IL‐2, interleukin‐2; PBMC, peripheral blood mononuclear cells; SARS‐CoV‐2, severe acute respiratory syndrome coronavirus 2

To distinguish between cross‐reactive memory T cells responding to peptides present within endemic HCoVs and SARS‐CoV‐2‐specific memory T cell responses, we designed a SARS‐CoV‐2‐specific peptide pool, herein referred to as TS16 (Sup. Table 1). In addition, a commercially available SARS‐CoV‐2 peptide pool, herein referred to as TB47 (Table S2), reported containing SARS‐CoV‐2‐specific epitopes was used. Memory T cell responses were determined using the Fluorospot (IFNγ and IL‐2) method. Both peptide pools generated a linear relationship between memory T cell production of IL‐2 and IFNγ (TB47; Spearman *r* = 0.9, TS16; Spearman *r*, 0.89, Figure 2A) displaying an equal response towards the two peptide pools. The peptide pools also demonstrated a similar linear relationship in terms of the number of IL‐2 producing memory T cells between the pools. However, the TB47 pool yielded increased levels of IFNγ compared with the TS16 pool (Figure 2B).

We next evaluated the proportion of study participants displaying a cellular response to the two SARS‐CoV‐2 peptide pools based on binary IFNγ readouts. We found that 93% of convalescent COVID‐19 patients (51/55) and 71% of anti‐S IgG positive HCW (155/216) displayed a SARS‐CoV‐2‐specific memory T cell response towards the TS16 pool 4–5 months post IgG seroconversion, while memory T cell responses were observed in only 4% of the anti‐S IgG negative HCW (5/115). Using the TB47 pool we found 96% of convalescent patients (53/55) and 86% of anti‐S IgG positive HCW (185/216) displayed SARS‐CoV‐2‐specific memory T cell responses. Interestingly, with the TB47 pool, up to 19% of anti‐S IgG negative HCW (22/115) displayed a positive memory T cell response (Figure 2C), not present with the in‐house designed TS16 pool. These seronegative individuals were not expected to have a memory response against neither of the two peptide pools, due to their serology status. Using dual S and N directed serology to identify a SARS‐CoV‐2 naïve and exposed group we have previously reported the specificity and sensitivity of each peptide pool and noted a high sensitivity but poor specificity with the commercial TB47 pool.^25^ This prompted us to determine the overlap of immunogenic regions between the two pools and endemic HCoV.

Peptides with sequences overlapping with the proteome of endemic HCoVs included in the two peptide pools have previously been reported in Mangsbo et al.,^25^ and are also summarized in Table S3. The TS16 pool contains no more than five amino acid sequences matching endemic HCoVs. The TB47 pool however contains multiple longer peptide matches arising from the N‐protein with one specific peptide identified with an 11 amino acid overlap and is contained within the LSPRWYFYYLGTGPEAGL sequence. An alignment of endemic HCoV regions towards this SARS‐CoV‐2 region is shown in Figure 2D and reveals a well‐conserved stretch of eight amino acids and the TCR exposed epitope as identified in the highlighted blue box in Figure 2D. Specific reactivity towards this sequence has been reported in COVID‐19 convalescent and uninfected individuals.^26^, ^27^ Our in silico HLA prediction shows that this peptide binds a wide range of HLA Class II molecules. Thus, the memory T cell responses to this sequence in seronegative individuals possibly originate from exposure to a previous endemic HCoV infection,^19^, ^27^ influencing the analysis of samples from individuals who have had previous HCoV infection.

### 3.2 The magnitude of long‐term SARS‐CoV‐2‐specific memory T cell responses correlate with long‐term SARS‐CoV‐2‐specific humoral immune responses and disease severity

**Figure 3:**
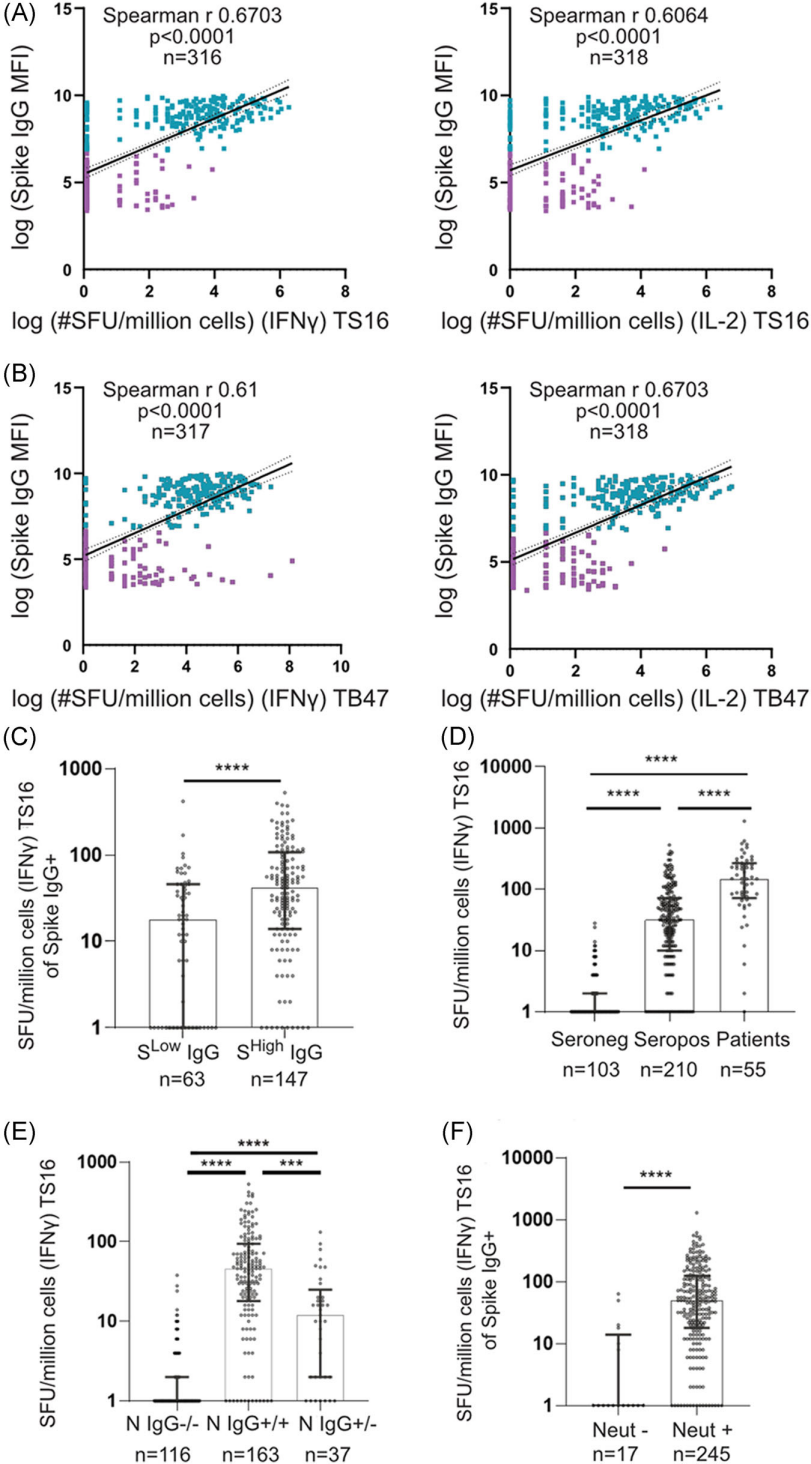
The magnitude and quantity of antibody responses are linked to memory T cell responses. (A and B) HCW IFNγ (left) and IL‐2 secreting (right) SFU/million cells correlated to circulating Spike IgG MFI levels measured at the same time point as PBMC collection. Linear regression with 95% confidence intervals displayed. Blue = anti‐Spike IgG^+^. Purple = anti‐Spike IgG^−^ (C) IFNγ SFU/million cells stimulated with TS16. Seropositive HCW (anti‐Spike IgG^+^), separated by Spike^Low^ (MFI < HCW average) or Spike^High^ (MFI > HCW average) measured at the time of PBMC collection. (D) IFNγ SFU/million cells stimulated with TS16. Seronegative HCW are Spike IgG^‐^ at all time‐points, seropositive HCW are continuous Spike IgG^+^ from study inclusion, and patients were admitted to the hospital with a confirmed COVID‐19 infection. (E) IFNγ SFU/million cells when stimulated with TS16 of HCW with a memory T cell response. Separated by Nucleocapsid IgG −/− (− at all time‐points), Nucleocapsid IgG + /+ (+ at all time‐points), Nucleocapsid IgG + /− (+ at study inclusion but ‐ at PBMC collection). (F) IFNγ SFU/million cells when stimulated with TS16 of HCW and patients (Spike IgG^+^ at study inclusion) with or without neutralizing antibodies. (A and B) see graphs for *n*, statistics and correlations were determined by Spearman *r* with a 95% confidence interval. Values were transformed with log(× + 1). (C–F) Median ± IQR displayed, for *n* see graphs. Statistics calculated by Mann–Whitney (C and F) or Kruskal–Wallis test and Dunn's test for multiple comparisons (D and E). ***p < .001, ****p < .0001. COVID‐19, coronavirus disease 2019; HCW, healthcare worker; IFN‐γ, interferon gamma; IQR, interquartile range; PBMC, peripheral blood mononuclear cells

We next investigated the relationship between SARS‐CoV‐2‐specific humoral immune responses and SARS‐CoV‐2‐specific cellular immune responses to the TS16 and TB47 pools 4–5 months post IgG seroconversion. Anti‐S IgG levels correlated well with the presence and magnitude of SARS‐CoV‐2‐specific memory T cell responses (Figure 3A,B). HCWs that displayed a lower MFI value of measured anti‐S IgG (below HCW average) demonstrated lower SARS‐CoV‐2‐specific memory T cell responses compared to those with a higher (above HCW average) anti‐S IgG response (Figure 3C). In concordance with previous reports^28^, ^29^ a high correlation between the anti‐S IgG and anti‐N IgG was identified (Figure S2A), and similar positive correlations were found between anti‐N IgG levels and SARS‐CoV‐2‐specific cellular immune responses of both TS16 and TB47 peptide pools (Figure S2B,C).

Previously hospitalized patients with severe COVID‐19 presented elevated levels of SARS‐CoV‐2‐specific T cell memory responses four months post IgG seroconversion compared to seropositive HCW with mild infection five months post IgG seroconversion (Figure 3D). Although the HCW samples were collected five months post IgG seroconversion as opposed to COVID‐19 patient samples that were collected four months post IgG seroconversion, the significant increases in SARS‐CoV‐2‐specific T cell responses found in COVID‐19 patients supports a link between disease severity and long‐term immunity. Lymphopenia has been reported during the acute phase of a SARS‐CoV‐2 infection, but white blood cell count, as well as lymphocyte counts, were equal between the HCW cohort and the patients as well as between seropositive and seronegative HCWs (Figure S3A) indicating that any impact that the SARS‐CoV‐2 infection has on circulating lymphocyte levels in the acute phase is transient, as has been noted by others.^30^ In addition, cellular responses to CMV in recovered individuals were identical between the cohorts, in line with a recent report ^30^ indicating that long‐term immunosenescence is not observed in response to a SARS‐CoV‐2 infection (Figure S3B).

Several groups have published data supporting that SARS‐CoV‐2 anti‐S IgG antibodies have a longer half‐life than anti‐N IgG.^30^, ^31^ Interestingly, HCW who displayed a decline in anti‐N IgG levels (n = 37), also displayed a significantly lower magnitude of SARS‐CoV‐2‐specific IFNγ responses compared to HCW who remained anti‐N IgG positive five months post IgG seroconversion (*n* = 163) (Figure 3E). SARS‐CoV‐2‐specific IFNγ responses 5 months post IgG seroconversion was furthermore elevated in participants with SARS‐CoV‐2 neutralizing antibodies measured four months post IgG seroconversion (Figure 3F), demonstrating that a robust memory T cell response is correlated with the generation of effectively neutralizing antibodies against SARS‐CoV‐2.

### 3.3 Selective COVID‐19 symptoms during the acute infection were associated with elevated long‐term SARS‐CoV‐2‐specific memory T cell responses

**Figure 4:**
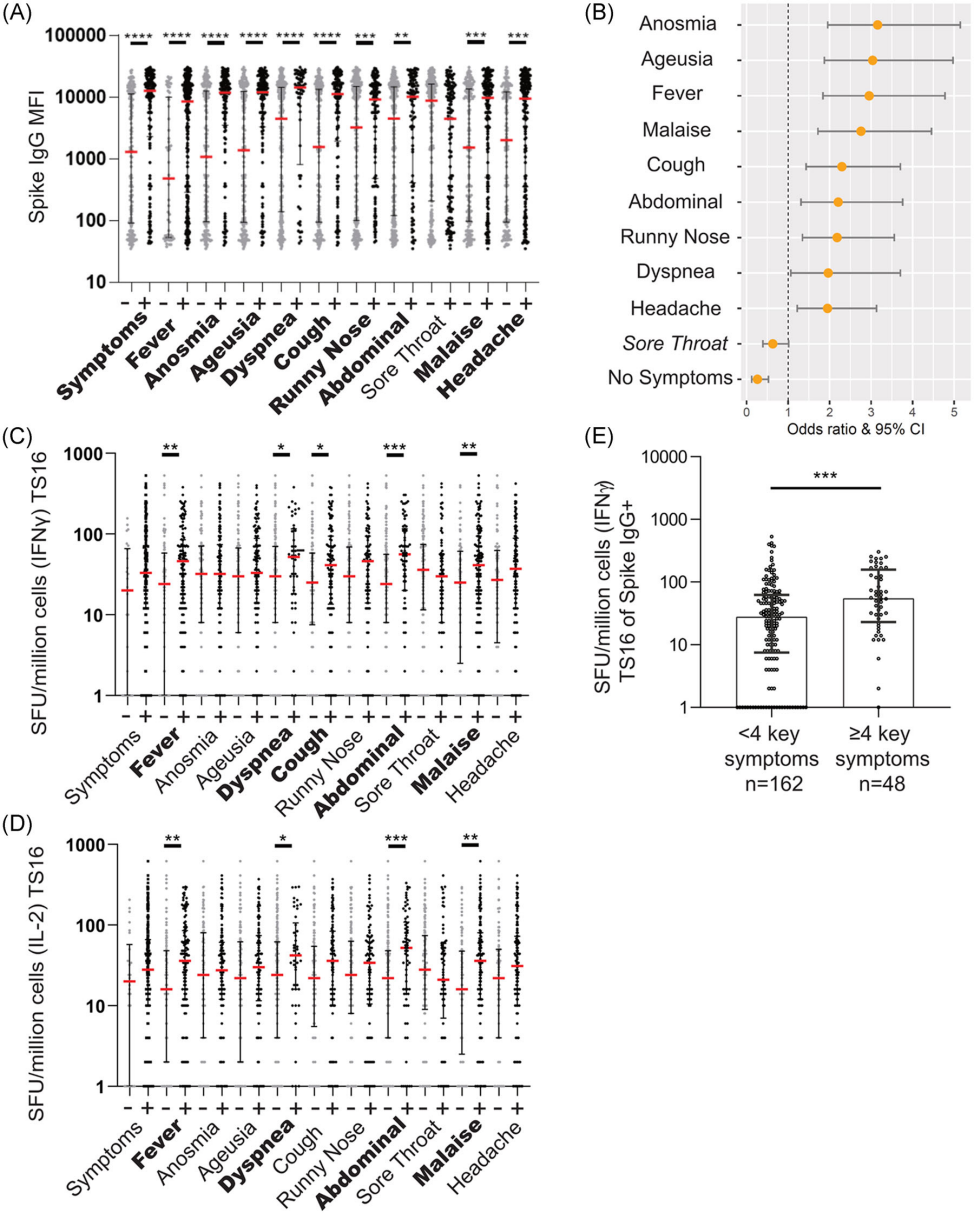
Symptoms of COVID‐19 are correlated with the presence and magnitude of memory immune responses. (A) Anti‐Spike IgG MFI levels from study inclusion in HCWs with (+) or without (‐) individual COVID‐19 specific symptoms during the period of possible initial infection. (B) Odds ratio scores for individual symptoms for all HCW to develop an IFNγ memory T cell response to the TS16 peptide pool. All symptoms were significant for correlation with memory response except those listed in italics. (C and D) IFNγ SFU/million cells or IL‐2 SFU/million cells when stimulated by TS16 peptide pool 5 months post IgG seroconversion. Seropositive HCW (Spike IgG^+^ all time‐points), separated by symptoms during the period of initial exposure. Symptoms with significantly higher levels of IFNγ or IL‐2 response are shown in bold. (E) IFNγ SFU/million cells when stimulated by TS16 of seropositive HCW five months post IgG seroconversion. Symptoms identified as significant in panel C are used to identify key symptoms. Separated by HCW having < 4 key symptoms or ≥4 key symptoms (A, C–E) median ± IQR displayed, *n* = 325 (A), *n* = 208 (C), *n* = 213 (D), see graph for *n* (E). Statistics calculated by Mann–Whitney. NS = not significant, *p < .05, **p < .01, ***p < .001, ****p < .0001. (B) Fisher's exact test was used to calculate the odds ratio (OR) with 95% confidence interval (CI). COVID‐19, coronavirus disease 2019; HCW, healthcare worker; IFN‐γ, interferon gamma; IQR, interquartile range

COVID‐19 symptomatology is highly heterogeneous but loss of anosmia and ageusia have shown to correlate with seroconversion.^22^ We noted that disease severity appeared to impact the magnitude of the cellular response (Figure 3D) and thus decided to investigate what self‐reported acute symptoms that correlate with the SARS‐CoV‐2‐specific cellular immune responses (TS16 peptide pool). First, we confirmed that this sub‐cohort (*n* = 388) of the larger COMMUNITY cohort (*n* = 2149) displayed similar serological alignment with symptoms previously reported.^22^ This was confirmed, as all donors that reported specific symptoms, except for sore throat, had a higher level of circulating anti‐S antibodies five months post IgG seroconversion than those that did not report the specific symptom (Figure 4A). High circulating antibody responses were however also seen in some asymptomatic HCW, suggesting that no individual symptom can predict if a person is likely to develop an antibody response.

Anosmia, ageusia, malaise, and fever were symptoms with the strongest association with maintaining a SARS‐CoV‐2‐specific memory T cell response five months post IgG seroconversion (Figure 4B) while sore throat had a negative correlation with a SARS‐CoV‐2‐specific memory T cell responses. The magnitude of SARS‐CoV‐2‐specific memory T cell responses will likely influence disease severity upon reinfection in cases where neutralization of the virus is not achieved. We identified four symptoms that correlate with a significant increase in the magnitude of IFNγ and IL‐2 producing SARS‐CoV‐2‐specific memory T cells: fever, dyspnea, abdominal symptoms, and malaise (+ cough for IFNγ) (Figure 4C,D). Examination of the magnitude of the SARS‐CoV‐2‐specific memory T cell response in HCW who had developed SARS‐CoV‐2‐specific anti‐S antibodies showed that individuals who reported multiple of these symptoms developed higher levels of SARS‐CoV‐2‐specific memory T cell responses 5 months post IgG seroconversion (Figure 4E), in support of that disease severity impacts the formation of a durable cellular memory response.

## 4 DISCUSSION

Investigations of cellular immune responses to SARS‐CoV‐2 have centered around the T cell response in the acute phase as well as studies focusing on phenotyping memory responses by cell surface markers and IFNγ release. Studies have reported declining circulating numbers of both total CD4^+^ and CD8^+^ T cells during the acute phase,^6^, ^7^, ^8^ while total CD4^+^ T cells decrease with disease severity.^32^ Published work has also demonstrated that memory T cells were detectable up to 80 days following severe disease and that the memory T cell response correlated to a B cell antibody response,^30^ while the prevalence of the memory immune response in patients with mild disease or over longer time‐points was not investigated. Most published literature is however biased contaminating measures of cross‐reactive cellular immune responses, and knowledge about the relationship between cellular and humoral responses and correlates to symptomology using a SARS‐CoV‐2‐specific peptide pool is lacking. In this study, we demonstrated that memory T cells remain detectable and correlate with humoral responses and disease severity in the majority of the study participants post mild to severe infection four to five months post IgG seroconversion (April–May 2020). Using an in‐house designed peptide pool that enabled identification of SARS‐CoV‐2‐specific memory T cells, we demonstrate that the memory T cell responses differ between subgroups of the disease, including individuals who lose anti‐N IgG responses over time, and those with severe disease or a subset of symptoms during the acute infection.

Even though patients with COVID‐19 develop detectable memory immune responses,^2^, ^30^, ^33^ it has been reported that individuals can become reinfected with SARS‐CoV‐2.^10^, ^11^, ^12^ In our study, we examined HCW with a range of disease symptoms, as well as hospitalized patients with severe disease. Overall, in this sub‐cohort study, 65% of the selected HCWs and 100% of patients with severe COVID‐19 were positive for anti‐S IgG when analyzed in spring 2020. The majority of these anti‐S IgG responses remained stable for up to 4–5 months (99%), however, 19% of HCW lost their anti‐N IgG and these individuals displayed inferior memory T cell responses 5 months post IgG seroconversion. These findings suggest that the majority of SARS‐CoV‐2 infections result in immune memory, though individuals that generate a poor memory T cell response were less likely to have generated long‐term anti‐N antibodies, which may be due to either dose or duration of viral exposure as well as host‐specific factors.

As previously mentioned, many commercial SARS‐CoV‐2 peptide pools display poor purity as well as overlapping antigen determinants to endemic HCoVs.^18^, ^20^, ^33^ In addition, studies have mainly focused on IFNγ as the main determinant for an effector response, skewing the measurement towards a specific memory T cell subset. Here, we examined both IL‐2 and IFNγ responses using a highly selective SARS‐CoV‐2‐specific peptide pool (TS16) without any identified overlapping T cell epitopes with endemic HCoV.^25^ Interestingly, the cross‐reactive response identified in SARS‐CoV‐2 seronegative HCWs was associated with increased IFNγ secretion, while SARS‐CoV‐2‐specific stimulation resulted in the release of both IL‐2 and IFNγ. This difference in the cytokine production profile could indicate different subsets of circulating memory T cells.^34^, ^35^, ^36^, ^37^, ^38^ An explanation to the IFNγ‐dominant secretion profile could be a biased effector memory T cell (TEM) phenotype, while SARS‐CoV‐2‐specific memory T cells may be of a central memory T cell (TCM) phenotype consistent with IL‐2 production or polyfunctional capacity associated with TCM.^38^, ^39^ Polyfunctional memory T cells have been shown to generate improved recall responses following vaccination.^40^ In work by Weiskopf et al.^41^ TCM cells responsive to SARS‐CoV‐2 peptides were identified in a small cohort, supporting our results. Alternative explanations for these results could be cross‐reactive responses of a lower avidity,^42^ or recent re‐exposure to the virus. Other studies using peptides that overlap with identified epitopes in HCoV, have measured memory T cell IFNγ production,^33^, ^43^ but not reported on IL‐2 production in these cells.

Limitations to our study include gender‐biased cohorts, due to population differences in the patients and HCW, and the subjective nature of self‐reported symptoms as well as lack of initial PCR testing. In addition, despite the time difference in the PBMC sampling of the HCW and patients, the memory response is a 4.6‐fold increase in the median between the seropositive HCW and the patients. However, the large difference is not likely to depend on one month difference in time span since infection. Additionally, the timeframe postinfection ranges from four‐six months, as we do not know the exact dates of infection. Based on the predicted HLA‐coverage, high sensitivity calculated from the patient population,^25^ and class epitope density for the peptides included in the in‐house peptide pool, HLA coverage is not likely to be a major limitation. Our large and well‐documented cohort was utilized to examine if symptoms affected the levels of cellular immune responses, with the use of a SARS‐CoV‐2‐specific peptide pool. We have identified that SARS‐CoV‐2‐specific memory T cell responses generate both IL‐2 and IFNγ memory T cell responses following mild to severe COVID‐19, correlating with long‐lasting neutralizing antibody responses. Additionally, we found that cross‐reactive memory T cells are skewed towards an IFNγ response. Furthermore, we identified five key symptoms (fever, dyspnea, cough, abdominal pain, or malaise) associated with an enhanced memory T cell response magnitude.

Our study suggests that through the course of natural infection, disease severity and specific symptoms influence the magnitude and possibly the duration of cellular immunity, which may impact long‐term immune protection. While this study has identified factors that can predict the level of the immune response, the pathophysiology behind these correlations, and whether they mirror the individual host susceptibility and overall viral load due to genetic or transcriptional host/organ variations, will be important areas for further research.

## CONFLICT OF INTERESTS

SM is the Chief Development Officer of Ultimovacs AB and is the founder and board member of Immuneed AB, Vivologica AB and Strike Pharma AB. IO, ML, ME, and PD are founders of Strike pharma. PD is the founder and employee of Scicross AB. None of the companies have any interest in the subject matter and have not taken part in the described work presented herein. None of the other authors declare any conflict of interest.

## ETHICS STATEMENT

The study applies with the declaration of Helsinki, and all study participants provided informed consent. The study is approved by the Swedish Ethical Review Authority (2020‐01653).

## AUTHOR CONTRIBUTION

*Conceptualization*: Jonas Klingström, Sophia Hober, Peter Nilsson, Mia Philipson, Pierre Dönnes, Sara Mangsbo, and Charlotte Thålin. *Methodology*: Ida Laurén, Sebastian Havervall, Henry Ng, Martin Lord, Aleksandra Pettke, Joachim Burman, Feifei Xu, Elisa Pin, Anna Månberg, Jonas Klingström, and Gustaf Christoffersson, Sophia Hober, Peter Nilsson, Mia Philipson, Pierre Dönnes, Robin Lindsay, Sara Mangsbo, and Charlotte Thålin. *Software*: Ida Laurén, Sophia Hober, Henry Ng, Martin Lord, Pierre Dönnes, and Robin Lindsay. *Validation*: Ida Laurén, Sebastian Havervall, Henry Ng, Martin Lord, Feifei Xu, Elisa Pin, Anna Månberg, Jonas Klingström, Gustaf Christoffersson, Sophia Hober, Peter Nilsson, Mia Philipson, Pierre Dönnes, Robin Lindsay, Sara Mangsbo, and Charlotte Thålin. *Formal analysis*: Ida Laurén, Sebastian Havervall, Henry Ng, Martin Lord, August Jernbom Falk, Wanda Christ, Anna Wiberg, My Hedhammar, Hanna Tegel, Joachim Burman, Feifei Xu, Elisa Pin, Anna Månberg, Jonas Klingström, Gustaf Christoffersson, Sophia Hober, Peter Nilsson, Mia Philipson, Pierre Dönnes, Robin Lindsay, Sara Mangsbo, and Charlotte Thålin. *Investigation*: Ida Laurén, Sebastian Havervall, Henry Ng, Martin Lord, Aleksandra Pettke, Nina Greilert‐Norin, Lena Gabrielsson, Aikaterini Chourlia, Catarina Amoêdo‐Leite, Vijay Sai Josyula, Mohamed Eltahir, Iliana Kerzeli, August Jernbom Falk, Jonathan Hober, Wanda Christ, Anna Wiberg, My Hedhammar, Hanna Tegel, Joachim Burman, Feifei Xu, Elisa Pin, Anna Månberg, Jonas Klingström, Gustaf Christoffersson, Sophia Hober, Peter Nilsson, Mia Philipson, Pierre Dönnes, Robin Lindsay, Sara Mangsbo, and Charlotte Thålin. *Data Curation*: Ida Laurén, Sebastian Havervall, Henry Ng, Martin Lord, August Jernbom Falk, Feifei Xu, Elisa Pin, Anna Månberg, Jonas Klingström, Gustaf Christoffersson, Sophia Hober, Peter Nilsson, Mia Philipson, Pierre Dönnes, Robin Lindsay, Sara Mangsbo, and Charlotte Thålin. *Writing – Original draft* – Ida Laurén, Sebastian Havervall, Martin Lord, Mia Philipson, Pierre Dönnes, Robin Lindsay, Sara Mangsbo, and Charlotte Thålin. *Writing – Review & Editing*: Ida Laurén, Sebastian Havervall, Henry Ng, Martin Lord, Aleksandra Pettke, Nina Greilert‐Norin, Lena Gabrielsson, Aikaterini Chourlia, Catarina Amoêdo‐Leite, Vijay Sai Josyula, Mohamed Eltahir, Iliana Kerzeli, August Jernbom Falk, Jonathan Hober, Wanda Christ, Anna Wiberg, My Hedhammar, Hanna Tegel, Joachim Burman, Feifei Xu, Elisa Pin, Anna Månberg, Jonas Klingström, Gustaf Christoffersson, Sophia Hober, Peter Nilsson, Mia Philipson, Pierre Dönnes, Robin Lindsay, Sara Mangsbo, and Charlotte Thålin. *Visualization*: Ida Laurén, Martin Lord, Pierre Dönnes, Robin Lindsay, Sara Mangsbo, and Charlotte Thålin. *Supervision*: Jonas Klingström, Gustaf Christoffersson, Sophia Hober, Peter Nilsson, Mia Philipson, Pierre Dönnes, Robin Lindsay, Sara Mangsbo, and Charlotte Thålin. *Project Administration*: Ida Laurén, Sebastian Havervall, Robin Lindsay, Sara Mangsbo, and Charlotte Thålin. *Funding Acquisition*: Jonas Klingström, Gustaf Christoffersson, Sophia Hober, Peter Nilsson, Mia Philipson, Sara Mangsbo, and Charlotte Thålin.

## Supporting information

Supplementary figure 1.  **HLA coverage of TS16 pool**. (A and B) World population coverage of the selected HLA I and HLA II alleles used for the *in silico* SARS‐CoV‐2 T cell epitope prediction and peptide selection. The TS16 pool encompasses T cell epitopes predicted to bind to respective HLA class I and HLA class II alleles. C) Total HLA coverage for the combined HLA I and HLA II alleles. The x‐axis depicts the frequency of individuals in the world carrying one or more of the HLA I and HLA II alleles covered by the SARS‐CoV‐2 T cell epitopes present in the TS16 pool. The corresponding cumulative percentage is detailed in red above.

Supplementary figure 2.  **Nucleocapsid IgG is correlated to T cell response. (A) HCW correlation between Spike IgG MFI and Nucleocapsid IgG MFI four to five months post study inclusion**. B and C) HCW IFNγ (left) and IL‐2 (right) SFU/million cells correlated to circulating Nucleocapsid IgG four to five months post study inclusion. (A‐C) Linear regression with 95% confidence intervals displayed. Blue = binary Spike IgG^+^. Purple = binary Spike IgG^‐^. n = 316‐322, statistics and correlations were determined by Spearman r with a 95% confidence interval. Values were transformed with log(x + 1).

Supplementary figure 3.  **WBC, Lymphocyte, and T cell response to CMV are not affected by COVID‐19 infection long‐term**. (A) White blood cell (WBC) and lymphocyte counts are shown for HCW (seropositive or seronegative at all time points), and hospitalized patients four to five months post study inclusion. B) IFNγ SFU/million cells in response to stimulation with a CMV peptide pool. A‐B) Median + /‐ IQR displayed, for n see graphs. Statistics calculated by Kruskal‐Wallis test and Dunn's test for multiple comparisons. NS = not significant.

Supporting information.

## Data Availability

Data is available upon request if within legal and personal protection boundaries.
