# Supplementary figures and images for: Long‐term SARS‐CoV‐2‐specific and cross‐reactive cellular immune responses correlate with humoral responses, disease severity, and symptomatology
Source: Immun Inflamm Dis. 2022 Mar 14;10(4):e595. doi: 10.1002/iid3.595 (PMC8962644; doi:10.1002/iid3.595)

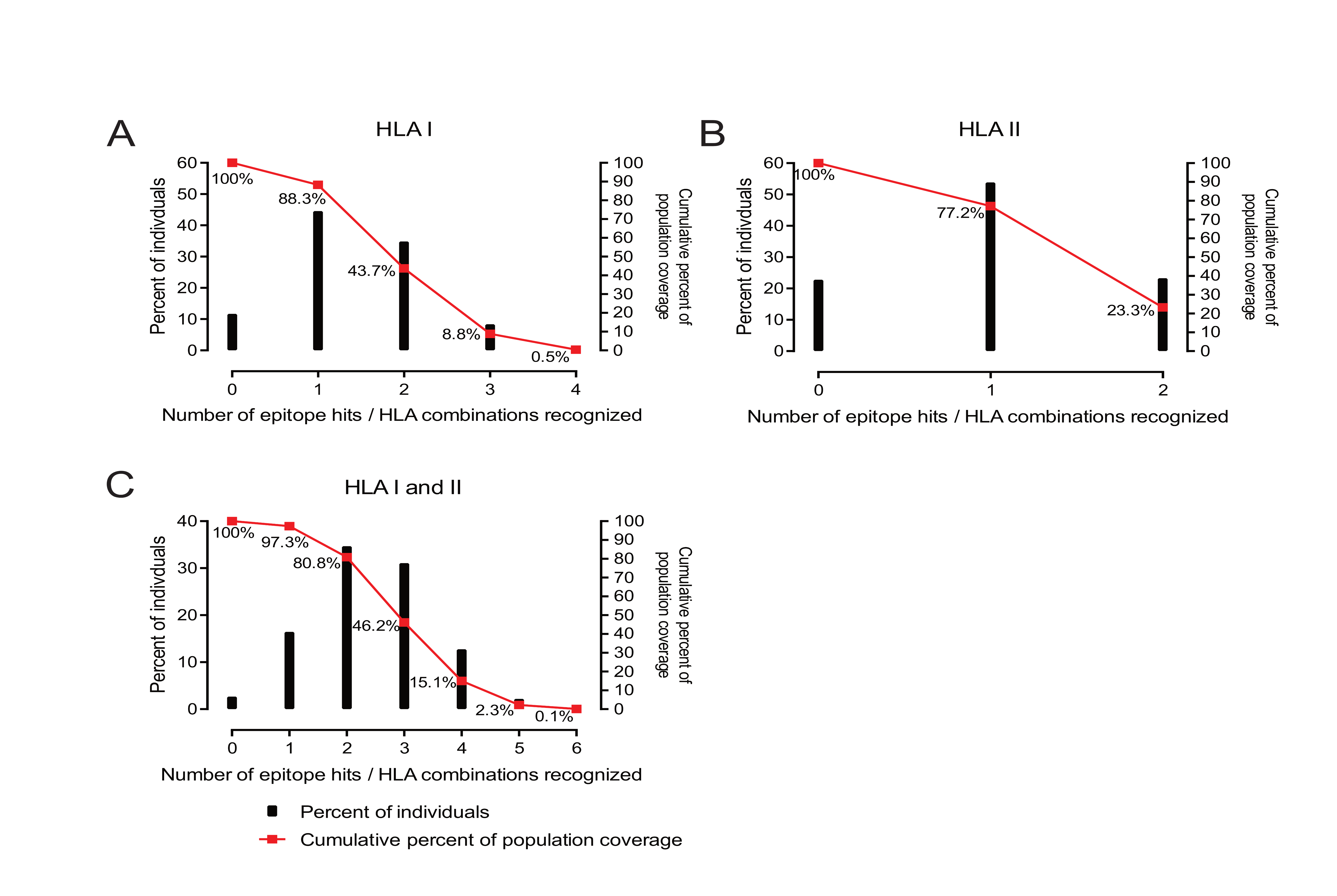

Supplement: Supplementary file 1 — Supplementary figure 1. HLA coverage of TS16 pool. (A and B) World population coverage of the selected HLA I and HLA II alleles used for the in silico SARS‐CoV‐2 T cell epitope prediction and peptide selection. The TS16 pool encompasses T cell epitopes predicted to bind to respective HLA class I and HLA class II alleles. C) Total HLA coverage for the combined HLA I and HLA II alleles. The x‐axis depicts the frequency of individuals in the world carrying one or more of the HLA I and HLA II alleles covered by the SARS‐CoV‐2 T cell epitopes present in the TS16 pool. The corresponding cumulative percentage is detailed in red above. [file IID3-10-0-s004.tiff]

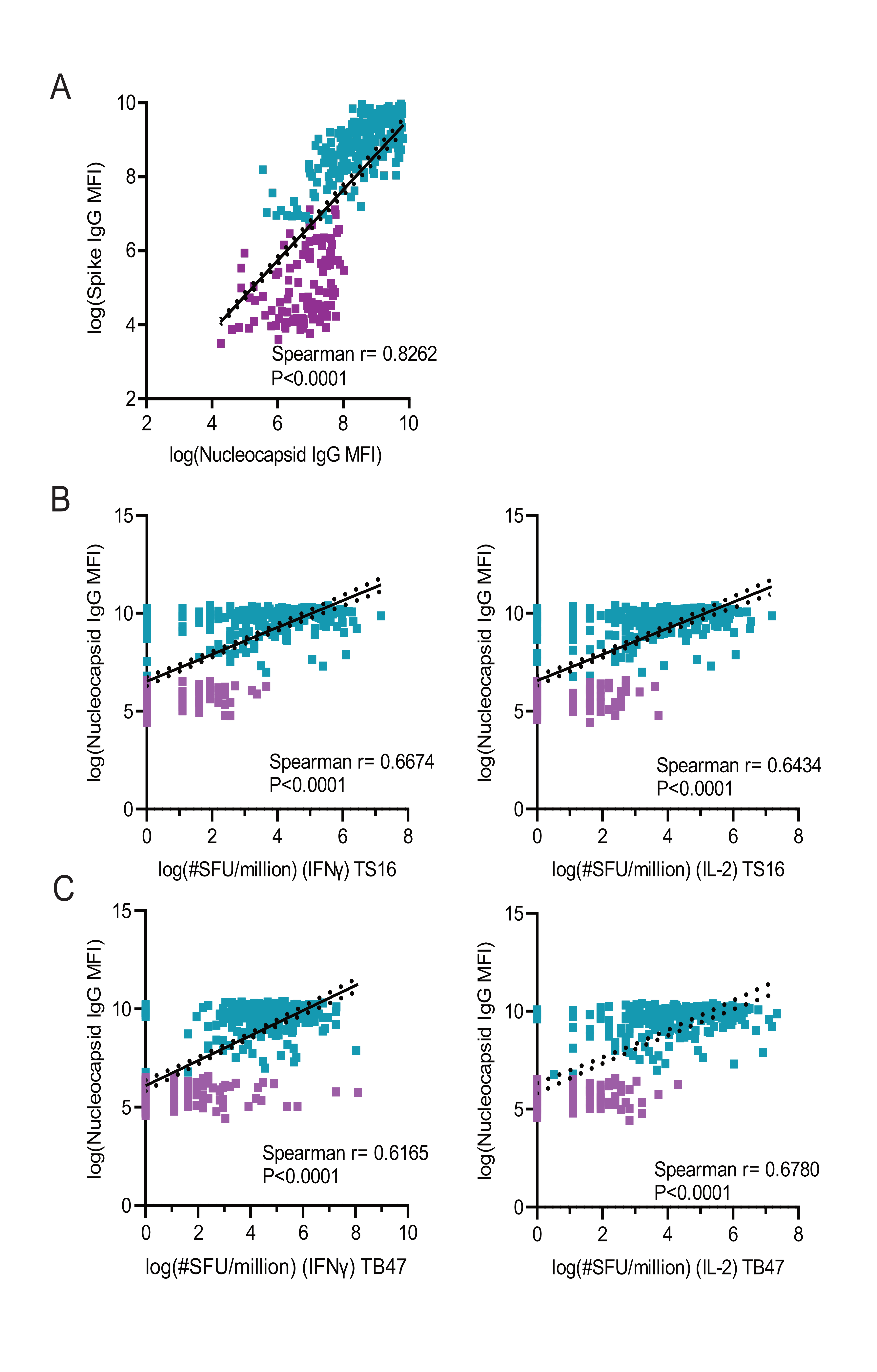

Supplement: Supplementary file 2 — Supplementary figure 2. Nucleocapsid IgG is correlated to T cell response. (A) HCW correlation between Spike IgG MFI and Nucleocapsid IgG MFI four to five months post study inclusion. B and C) HCW IFNγ (left) and IL‐2 (right) SFU/million cells correlated to circulating Nucleocapsid IgG four to five months post study inclusion. (A‐C) Linear regression with 95% confidence intervals displayed. Blue = binary Spike IgG+. Purple = binary Spike IgG‐. n = 316‐322, statistics and correlations were determined by Spearman r with a 95% confidence interval. Values were transformed with log(x + 1). [file IID3-10-0-s001.tiff]

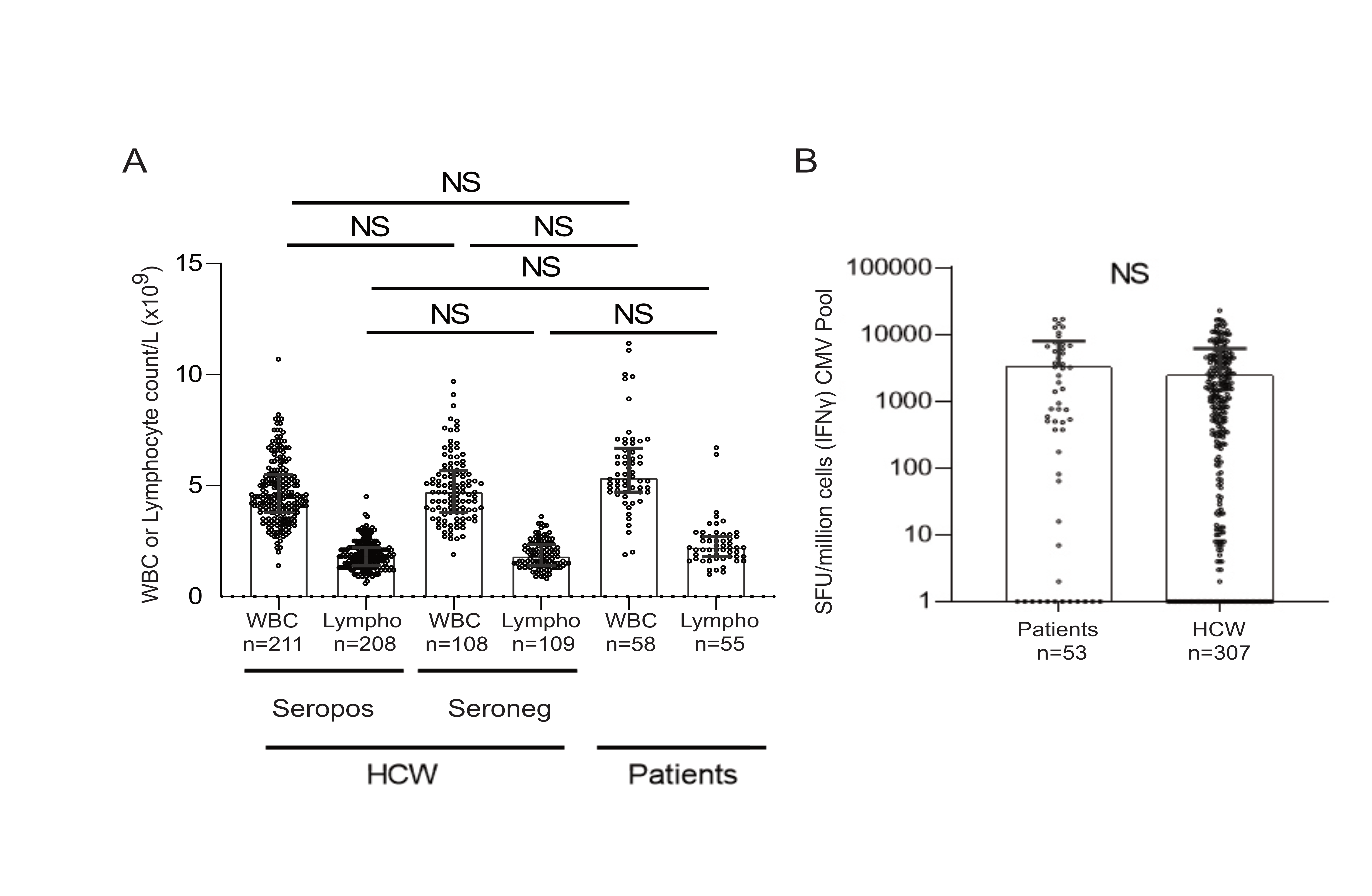

Supplement: Supplementary file 3 — Supplementary figure 3. WBC, Lymphocyte, and T cell response to CMV are not affected by COVID‐19 infection long‐term. (A) White blood cell (WBC) and lymphocyte counts are shown for HCW (seropositive or seronegative at all time points), and hospitalized patients four to five months post study inclusion. B) IFNγ SFU/million cells in response to stimulation with a CMV peptide pool. A‐B) Median + /‐ IQR displayed, for n see graphs. Statistics calculated by Kruskal‐Wallis test and Dunn's test for multiple comparisons. NS = not significant. [file IID3-10-0-s002.tiff]
