# Supplementary material for: Long‐term SARS‐CoV‐2‐specific and cross‐reactive cellular immune responses correlate with humoral responses, disease severity, and symptomatology
Source: Immun Inflamm Dis. 2022 Mar 14;10(4):e595. doi: 10.1002/iid3.595 (PMC8962644; doi:10.1002/iid3.595)
Supplement: Supplementary file 4 — Supporting information. [file IID3-10-0-s003.docx]

|  | Peptide sequence in TS16 pool | Protein | DRB1*  101 | DRB1*  301 | DRB1*  401 | DRB1*  701 | DRB1*  801 | DRB1*  1101 | DRB1*  1301 | DRB1*  1501 | A*02:01 | A*01:01 | A*03:01 | A*11:01 | A*24:02 | B*07:02 | B*08:01 | B*40:01 |
| --- | --- | --- | --- | --- | --- | --- | --- | --- | --- | --- | --- | --- | --- | --- | --- | --- | --- | --- |
| 1 | GAAAYYVGYLQPRTFLLKYNENGTI | S protein | 0 | 0 | 1 | 1 | 0 | 0 | 1 | 0 | 1 | 1 | 2 | 1 | 5 | 4 | 4 | 0 |
| 2 | YSVLYNSASFSTFKCYGVSPTKLNDLCFTN | S protein | 1 | 0 | 1 | 1 | 0 | 1 | 1 | 2 | 1 | 1 | 3 | 3 | 3 | 1 | 1 | 0 |
| 3 | NYLYRLFRKSNLKPFERDIS | S protein | 0 | 0 | 0 | 0 | 0 | 2 | 2 | 0 | 0 | 0 | 2 | 2 | 1 | 0 | 4 | 0 |
| 4 | YGFQPTNGVGYQPYRVVVLS | S protein | 2 | 0 | 1 | 3 | 0 | 0 | 0 | 1 | 1 | 0 | 0 | 1 | 2 | 2 | 2 | 1 |
| 5 | FQQFGRDIADTTDAVRDPQTLEILD | S protein | 0 | 1 | 0 | 0 | 1 | 1 | 1 | 0 | 1 | 0 | 1 | 1 | 0 | 4 | 1 | 1 |
| 6 | NQVAVLYQGVNCTEVPV | S protein | 0 | 0 | 0 | 1 | 1 | 0 | 0 | 0 | 2 | 0 | 0 | 1 | 0 | 0 | 0 | 0 |
| 7 | NQVAVLYQDVNCTEVPV | S protein | 0 | 1 | 0 | 0 | 0 | 0 | 1 | 0 | 1 | 0 | 0 | 1 | 0 | 0 | 0 | 0 |
| 8 | CTFEYVSQPFLMDLE | S protein | 0 | 0 | 1 | 1 | 1 | 0 | 0 | 0 | 0 | 0 | 0 | 0 | 2 | 2 | 0 | 0 |
| 9 | GLPNNTASWFTALTQHGKEDL | N protein | 1 | 0 | 1 | 0 | 1 | 1 | 0 | 0 | 1 | 0 | 0 | 2 | 1 | 2 | 1 | 0 |
| 10 | IIWVATEGALN | N protein | 2 | 0 | 1 | 0 | 1 | 0 | 0 | 0 | 0 | 0 | 0 | 0 | 0 | 1 | 0 | 0 |
| 11 | GPQNQRNAPRITFGGPS | N protein | 0 | 0 | 0 | 0 | 0 | 0 | 0 | 0 | 0 | 0 | 1 | 0 | 1 | 2 | 0 | 0 |
| 12 | GPQNQRNALRITFGGPS | N protein | 0 | 0 | 0 | 1 | 0 | 0 | 0 | 1 | 0 | 0 | 0 | 0 | 1 | 1 | 1 | 0 |
| 13 | GAVILRGHLRIAGHHLGR | M | 1 | 0 | 0 | 0 | 1 | 1 | 0 | 0 | 2 | 0 | 1 | 2 | 1 | 1 | 3 | 0 |
| 14 | TSRTLSYYKLGASQRVA | M | 2 | 0 | 0 | 2 | 0 | 0 | 0 | 1 | 0 | 0 | 1 | 0 | 1 | 0 | 1 | 0 |
| 15 | FASFYYVWKSYVHVVD | NSP3 | 0 | 0 | 1 | 2 | 0 | 0 | 0 | 0 | 2 | 0 | 1 | 2 | 2 | 0 | 2 | 0 |
| 16 | HNDILLAKDTTEAFE | NSP7 | 0 | 1 | 1 | 0 | 0 | 0 | 1 | 0 | 1 | 0 | 0 | 0 | 0 | 0 | 2 | 0 |

Supplementary Table 1. Peptide sequences of the TS16 peptide pool and identified numbers of HLA class I and HLA class II epitopes.

Supplementary Table 2. Peptide sequences of the TB47 peptide pool.

| Peptide number | Peptide sequence | Protein |
| --- | --- | --- |
| 1 | CTFEYVSQPFLMDLE | Spike |
| 2 | EFVFKNIDGYFKIYS | Spike |
| 3 | KHTPINLVRDLPQGF | Spike |
| 4 | NLVRDLPQGFSALEP | Spike |
| 5 | YAWNRKRISNCVADY | Spike |
| 6 | GVSPTKLNDLCFTNV | Spike |
| 7 | GGNYNYLYRLFRKSN | Spike |
| 8 | YLYRLFRKSNLKPFE | Spike |
| 9 | VVLSFELLHAPATVC | Spike |
| 10 | GPKKSTNLVKNKCVN | Spike |
| 11 | SVTTEILPVSMTKTS | Spike |
| 12 | STECSNLLLQYGSFC | Spike |
| 13 | NLLLQYGSFCTQLNR | Spike |
| 14 | NFSQILPDPSKPSKR | Spike |
| 15 | TDEMIAQYTSALLAG | Spike |
| 16 | GINASVVNIQKEIDR | Spike |
| 17 | LIDLQELGKYEQYI | Spike |
| 18 | YEQYIKWPWYIWLGF | Spike |
| 19 | MSDNGPQNQRNAPRITF | Nucleoprotein |
| 20 | NQRNAPRITFGGPSDSTG | Nucleoprotein |
| 21 | DQIGYYRRATRRIR | Nucleoprotein |
| 22 | MKDLSPRWYFYYL | Nucleoprotein |
| 23 | LSPRWYFYYLGTGPEAGL | Nucleoprotein |
| 24 | AFFGMSRIGMEVTPSGTW | Nucleoprotein |
| 25 | GMEVTPSGTWLTYTGAIK | Nucleoprotein |
| 26 | TWLTYTGAIKLDDKDPNF | Nucleoprotein |
| 27 | PNFKDQVILLNKHIDAYK | Nucleoprotein |
| 28 | LLNKHIDAYKTFPPTEPK | Nucleoprotein |
| 29 | LLESELVIGAVILRGHLR | Membrane protein |
| 30 | GAVILRGHLRIAGHHLGR | Membrane protein |
| 31 | LRIAGHHLGRCDIKDLPK | Membrane protein |
| 32 | PKEITVATSRTLSYYKL | Membrane protein |
| 33 | TSRTLSYYKLGASQRVA | Membrane protein |
| 34 | IGNYKLNTDHSSSSDNIA | Membrane protein |
| 35 | YFLCWHTNCYDYCIPY | ORF 3a |
| 36 | KDCVVLHSYFTSDYYQLY | ORF 3a |
| 37 | YFTSDYYQLYSTQLSTDTGV | ORF 3a |
| 38 | GVEHVTFFIYNKIVDEPEEH | ORF 3a |
| 39 | LITLATCELYHYQECVR | ORF 7a |
| 40 | FHPLADNKFALTCFSTQF | ORF 7a |
| 41 | DGVKHVYQLRARSVSPKL | ORF 7a |
| 42 | ILLNKHID | Nucleoprotein |
| 43 | MEVTPSGTWL | Nucleoprotein |
| 44 | QLIRAAEIRASANLAATK | Spike |
| 45 | SPRWYFYYL | Nucleoprotein |
| 46 | YLGTGPEAGL | Nucleoprotein |
| 47 | YYLGTGPEA | Nucleoprotein |

Supplementary Table 3. Peptides with identified overlapping sequences with endemic HCoV within the TS16 and TB47 peptide pools. Indication of identified overlapping amino acids within the peptides are identified in bold.

| **Pool** | **Source protein** | **Peptide** | **Sequence** | **AA_a_** | **229E** | **OC43** | **HKU1** | **NL63** | **MERS** | **SARS-CoV** | **SARS-CoV-2** |
| --- | --- | --- | --- | --- | --- | --- | --- | --- | --- | --- | --- |
| **TS16** | Spike | YSVLYNSA**SFST**FKCYGVSPTKLNDLCFTN | SFSTF | 5 | X |  |  |  |  |  | X |
| **TS16** | Spike | YSVLYNSASFSTFKCYGVSPTKL**NDLCF**TN | NDLCF | 5 |  |  | X |  |  | X | X |
| **TS16** | NSP7 | **HNDIL**LAKDTTEAFE | HNDIL | 5 | X |  |  |  | X | X | X |
| **TB47** | Spike | GVSPTKL**NDLCF**TNV | NDLCF | 5 |  |  | X |  |  |  | X |
| **TB47** | Spike | STECSNLLLQ**YGSFC** | YGSFC | 5 |  | X |  |  |  | X | X |
| **TB47** | Spike | NLLLQ**YGSFC**TQLNR | YGSFC | 5 |  | X |  |  |  | X | X |
| **TB47** | Spike | TDEMIA**QYTSA**LLAG | QYTSA | 5 | X |  |  | X |  |  | X |
| **TB47** | Spike | YEQ**YIKWPW**YIWLGF | YIKWPW | 6 | X |  |  | X |  | X | X |
| **TB47** | Spike | YEQYI**KWPWY**IWLGF | KWPWY | 5 |  | X | X |  |  | X | X |
| **TB47** | Nucleoprotein | MKDLS**PRWYFYYL** | PRWYFYYL | 8 |  | X | X |  | X | X | X |
| **TB47** | Nucleoprotein | LS**PRWYFYYLGTG**PEAGL | PRWYFYYLGTG | 11 |  | X | X |  |  | X | X |
| **TB47** | Nucleoprotein | LSPRWY**FYYLGTG**PEAGL | FYYLGTG | 7 | X |  |  | X |  |  | X |
| **TB47** | Nucleoprotein | LLES**ELVIG**AVILRGHLR | ELVIG | 5 |  |  | X |  |  | X | X |
| **TB47** | ORF 3a | K**DCVVL**HSYFTSDYYQLY | DCVVL | 5 |  | X |  |  |  |  | X |
| **TB47** | ORF 3a | YFTSDYYQLYSTQLS**TDTGV** | TDTGV | 5 |  | X |  |  |  |  | X |
| **TB47** | Nucleoprotein | S**PRWYFYYL** | PRWYFYYL | 8 |  | X | X |  | X | X | X |
| **TB47** | Nucleoprotein | **YLGTGP**EAGL | YLGTGP | 6 | X | X | X | X |  | X | X |
| **TB47** | Nucleoprotein | **YYLGTGP**EA | YYLGTGP | 7 | X | X | X | X |  | X | X |

AA=amino acid
